# MiR-34a modulates ionizing radiation-induced senescence in lung cancer cells

**DOI:** 10.18632/oncotarget.19267

**Published:** 2017-07-15

**Authors:** Xiaoyuan He, Aimin Yang, Daniel G. McDonald, Ellen C. Riemer, Kenneth N. Vanek, Bradley A. Schulte, Gavin Y. Wang

**Affiliations:** ^1^ Department of Pathology and Laboratory Medicine, Medical University of South Carolina, Charleston, SC 29425, USA; ^2^ Department of Radiation Oncology, Medical University of South Carolina, Charleston, SC 29425, USA; ^3^ Cancer Genes and Molecular Regulation Program of Hollings Cancer Center, Medical University of South Carolina, Charleston, SC 29425, USA

**Keywords:** microRNA-34a (miR-34a), ionizing radiation, non-small cell lung cancer, cellular senescence, c-Myc

## Abstract

MicroRNAs (miRNAs) are a new class of gene expression regulators that have been implicated in tumorigenesis and modulation of the responses to cancer treatment including that of human non-small cell lung cancer (NSCLC). However, the role of miR-34a in ionizing radiation (IR)-induced senescence in NSCLC cells remains poorly understood. Here we report that IR-induced premature senescence correlates with upregulation of miR-34a expression in NSCLC cells. Ectopic overexpression of miR-34a by transfection with synthetic miR-34a mimics markedly enhances IR-induced senescence, whereas inhibition of miR-34a by transfection with a synthetic miR-34a inhibitor attenuates IR-induced senescence. Clonogenic assays reveal that treatment with miR-34a mimics augments IR-induced cell killing in human NSCLC cells. Mechanistically, we found that the senescence-promoting effect of miR-34a is associated with a dramatic down-regulation of c-Myc (Myc) expression, suggesting that miR-34a may promote IR-induced senescence via targeting Myc. In agreement with this suggestion, knockdown of Myc expression by RNAi recapitulates the senescence-promoting effect of miR-34a and enhances IR-induced cell killing in NSCLC cells. Collectively, these results demonstrate a previously unrecognized role for miR-34a in modulating IR-induced senescence in human NSCLC cells and suggest that pharmacological intervention of miR-34a expression may represent a new therapeutic strategy for improving the efficacy of lung cancer radiotherapy.

## INTRODUCTION

Lung cancer is the leading cause of cancer deaths both in the United States and worldwide [1]. Even with current advanced treatment, the 5-year overall survival rate is less than 16% and has not changed appreciably over many decades [1, 2]. This highly unsatisfactory clinical outcome emphasizes an urgent need for the development of novel therapeutic approaches to more effectively manage this deadly disease. One of the major reasons for the poor clinical outcome of lung cancer radiotherapy is likely due to the resistance of NSCLC cells to ionizing radiation (IR)-induced apoptosis [3, 4]. Cellular senescence suppresses cancer growth by permanently arresting the proliferation of oncogenic cells [5–7]. We and others have shown that the induction of senescence is an important mechanism underlying the tumor suppression effect of IR and certain chemotherapeutic agents [3, 5–10]. Notably, targeted cancer therapy and cancer immunotherapies have been shown to cause cancer regression via the induction of senescence in tumor cells [11, 12]. Moreover, our recent studies have demonstrated that activation of p53 by Nutilin-3 sensitizes NSCLC cells to radiation by enhancing IR-induced senescence [3], suggesting that pharmacological promotion of senescence induction can be exploited as a novel and effective therapeutic approach to improve the efficacy of lung cancer radiotherapy.

MicroRNAs (miRNAs) have emerged as a new class of modulators of gene expression and have been shown to be implicated in tumorigenesis, metastatic progression and therapeutic responses [13–18]. For example, it has been found that miR-34a, which can function as a tumor suppressor, is deregulated in many types of human cancers, including NSCLC [19–23]. Recently, our studies have demonstrated that a subset of miRNAs, including miR-34a, is involved in modulating IR-induced senescence [24]. However, it remains to be determined if miR-34a affects IR-induced senescence in human NSCLC cells. In the present study, we show that IR-induced senescence correlates well with up-regulation of miR-34a expression in human NSCLC cells. Mechanistically, we found that Myc is a target of miR-34a and that silencing of Myc by siRNA phenocopies the senescence-promoting effect of miR-34a, suggesting that miR-34a may promote IR-induced senescence in human NSCLC cells via targeting the Myc oncoprotein. These new findings strongly support the potential translational application of synthetic miR-34 mimics as a radiation sensitizer to improve the efficacy of lung cancer radiotherapy via augmenting IR-induced premature senescence.

## RESULTS

### IR induces premature senescence in human NSCLC cells in a dose-dependent manner

**Figure 1:**
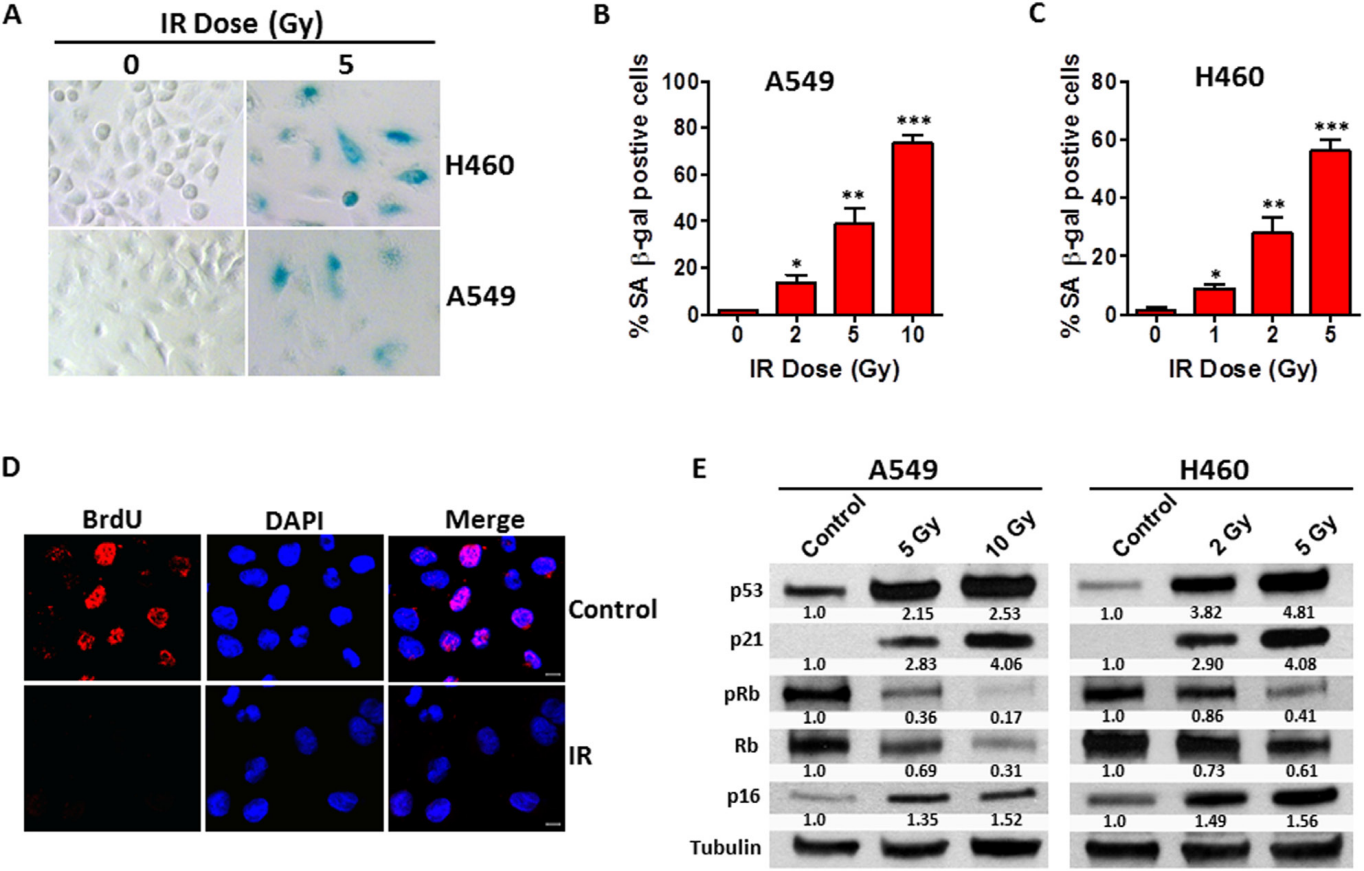
IR induces premature senescence in human NSCLC cells **(A)** SA-β-gal assays were performed 5 days after irradiation to identify senescent cells in irradiated NSCLC cells. Shown are representative microscopic images of SA-β-gal positive (Blue stained) senescent A549 and H460 cells. **(B)** The percentage of SA-β-gal positive senescent cells in irradiated and non-irradiated A549 cells was quantified and graphed. Data are presented as mean ± SEM of three independent assays. **(C)** The percentage of SA-β-gal positive senescent cells in irradiated and non-irradiated H460 cells is presented as mean ± SEM of three independent assays. **(D)** BrdU incorporation assays were performed 4 days after irradiation to determine proliferating versus senescent NSCLC cells. Representative images of confocal microscopic analysis of irradiated (5 Gy) H460 cells are shown. **(E)** Expression levels of p53, p21, pRb, Rb, and p16 in irradiated versus control NSCLC cells 4 days after irradiation were determined by Western blot analysis. Relative expression levels of immunoblot bands were quantified using Image J software and were normalized to the ratio of control cells. Tubulin was probed as a loading control. * p < 0.05 vs. control (0 Gy), ** p < 0.01 vs. control, *** p < 0.001 vs. control.

Increased senescence-associated β-galactosidase (SA-β-gal) activity is a hallmark of cellular senescence [25], thus SA-β-gal staining was performed to semi-quantify senescent cells in irradiated human NSCLC cells. As shown in Figures 1A–1C, the percentage of SA-β-gal positive senescent cells increased with IR dosage in both A549 and H460 cells, suggesting that IR induces premature senescence in human NSCLC cells in a dose-dependent fashion. Confocal microscopic analyses revealed that irradiated NSCLC cells almost completely lost their ability to incorporate BrdU (Figure 1D), confirming that they were in the state of permanent cell cycle arrest, a characteristic feature of cellular senescence. Moreover, cell growth curve studies showed that both A549 and H460 control cells grow exponentially while irradiated A549 and H460 cells lose their ability to proliferate in culture (Supplementary Figure 1).

To further verify the IR-induced senescence phenotype in NSCLC cells, we investigated the expression levels of a series of molecular markers for senescence [26], including p21, p16^*INK4*^ (p16) and phosphorylated Rb (pRb) levels in irradiated A549 and H460 cells. Immunoblotting assays indicated that the expression levels of p16 and p21 were increased significantly, whereas pRb levels declined markedly in irradiated NSCLC cells compared with non-irradiated control cells (Figure 1E). These data demonstrate that IR exposure is able to induce premature senescence in human NSCLC cells in a dose-dependent manner and that up-regulation of p21 and p16 expression as well as a decline in pRb levels are involved in IR-induced senescence in human NSCLC cells.

### IR-induced senescence correlates with up-regulation of miR-34a expression in NSCLC cells

**Figure 2:**
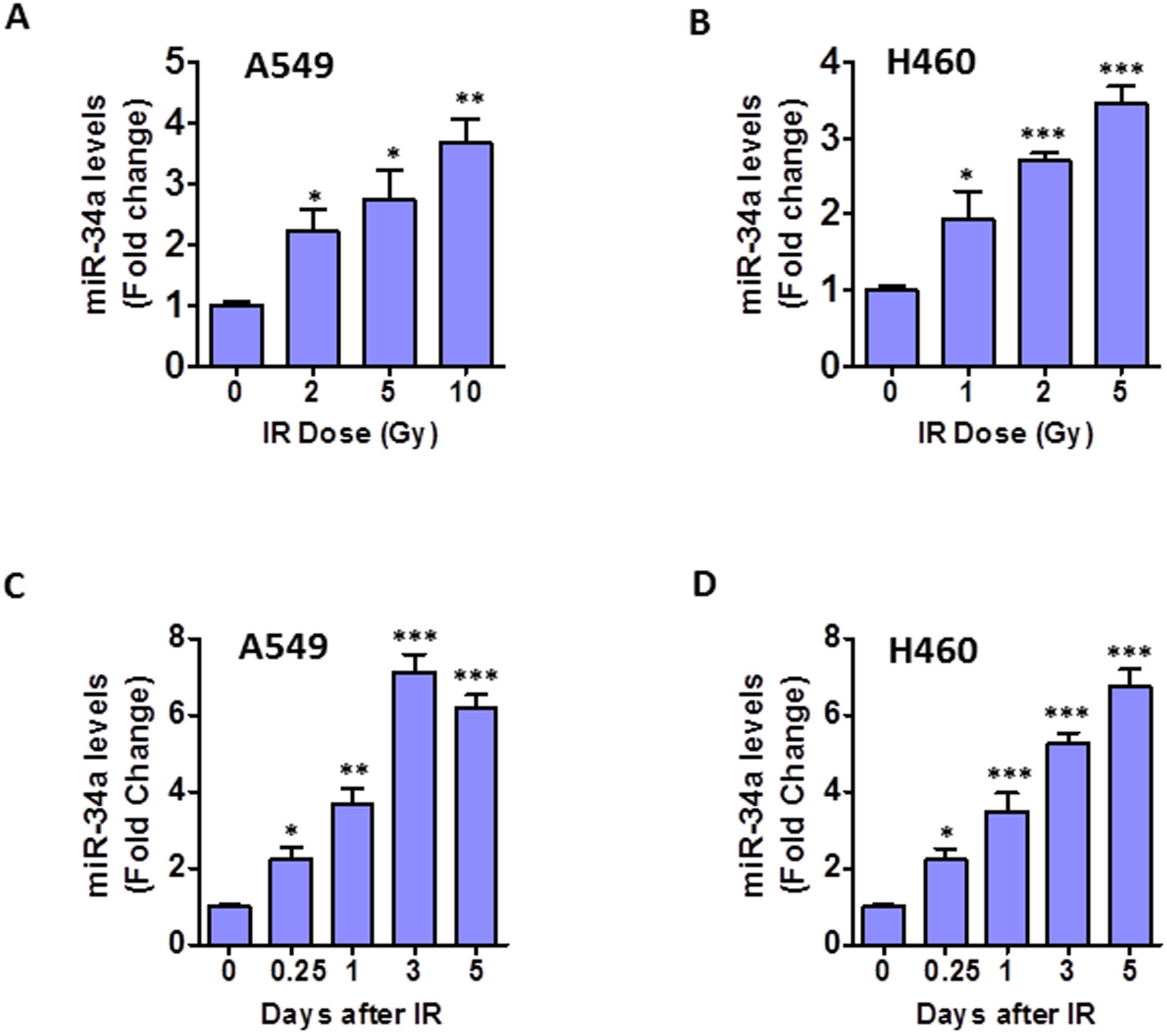
IR-induced senescence correlates with up-regulation of miR-34a in NSCLC cells **(A)** Expression levels of miR-34a in A549 cells were determined using TaqMan miRNA assays as we have reported previously [24] at 24 h after different doses of IR treatment. **(B)** Expression levels of miR-34a in H460 cells were determined using TaqMan miRNA assays at 24 h after different doses of IR exposure. **(C, D)** Shown are miR-34a expression levels at different time points after irradiation of A549 (10 Gy) and H460 (5 Gy) cells. Data are presented as mean ± SEM of three independent experiments. * p < 0.05 vs. control (0 Gy), ** p < 0.01 vs. control, *** p < 0.001 vs. control.

Our recent studies have showed that miR-34a is involved in IR-induced premature senescence in human lung fibroblasts [24]. However, the role of miR-34a in IR-induced senescence in human NSCLC cells is largely unknown. To address this issue, we examined miR-34a expression levels in response to different doses of irradiation. TaqMan miRNA assays revealed that miR-34a expression levels increased substantially with IR dosage in both A549 and H460 cells (Figures 2A & 2B). Furthermore, time course studies indicated that miR-34a expression levels in irradiated A549 cells were elevated in a time-dependent manner, reached peak levels at 3 days after IR and persisted at a high levels even 5 days after IR exposure (Figure 2C). Similarly, a time-dependent increase of miR-34a expression was observed in irradiated H460 cells (Figure 2D). These findings demonstrate for the first time that IR-induced senescence correlates with persistent up-regulation of miR-34a in NSCLC cells, suggesting a role of miR-34a in modulating IR-induced senescence in lung cancer cells.

### Ectopic overexpression of miR-34a enhances IR-induced senescence in NSCLC cells

**Figure 3:**
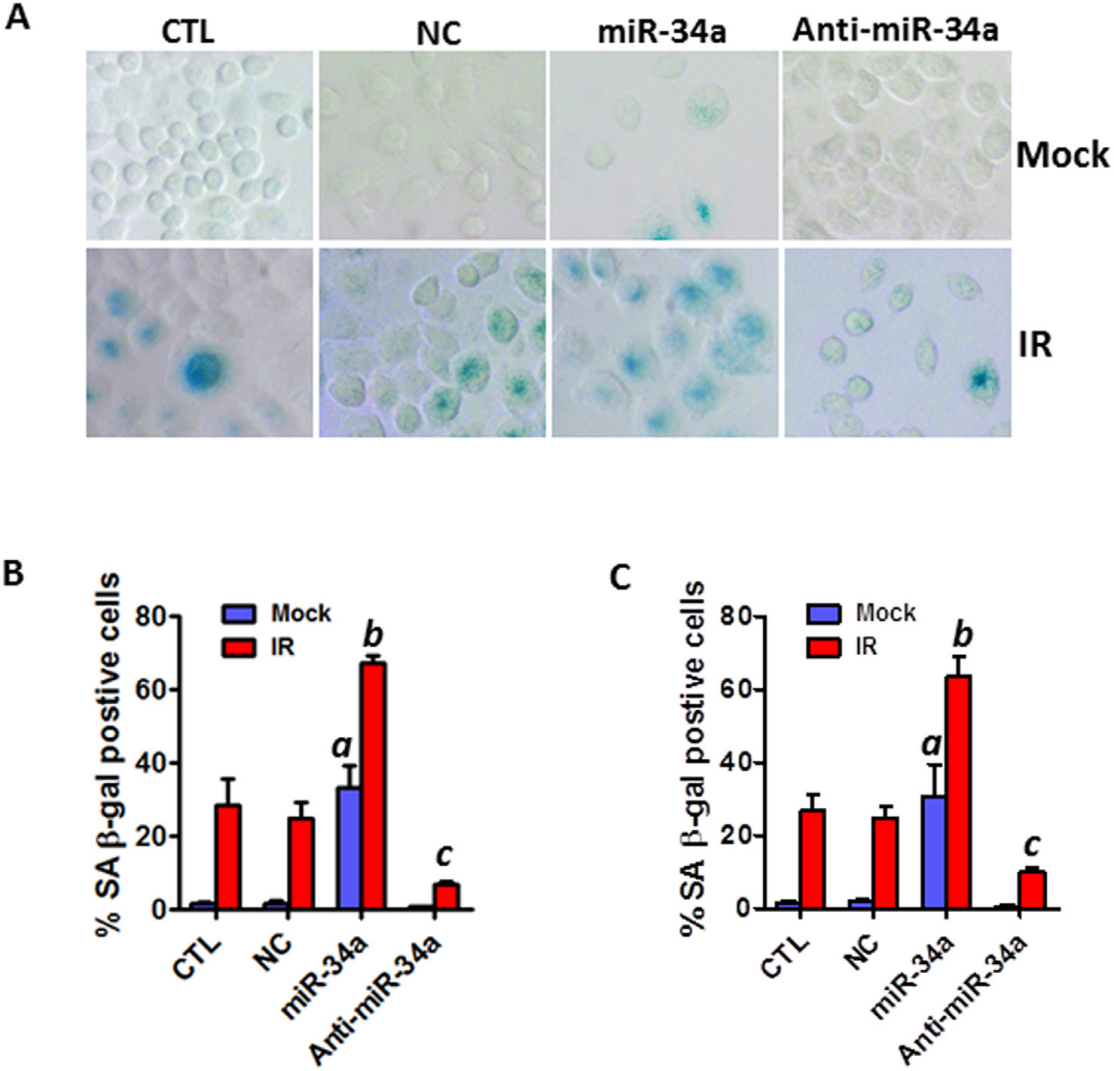
Ectopic overexpression of miR-34a enhances IR-induced premature senescence in NSCLC cells **(A)** SA-β-gal staining was employed to identify senescent cells in irradiated and non-irradiated (mock) NSCLC cells 6 days after miR-34a mimic (miR-34a) or miR-34a inhibitor (Anti-miR-34a) transfection. **(B)** The percentage of SA-β-gal positive senescent cells in irradiated (2 Gy) and control H460 cells is presented as mean ± SEM of three independent assays. **(C)** The percentage of SA-β-gal positive senescent cells in irradiated (5 Gy) and control A549 cells is presented as mean ± SEM of three independent assays. a, p < 0.01 vs. control; b, p < 0.001 vs. NC + IR; c, p < 0.001 vs. NC + IR.

To investigate a causal role of miR-34a in IR-induced senescence in NSCLC cells, we examined if overexpression of miR-34a by transfection with synthetic miR-34a mimics affects IR-induced senescence in A549 and H460 cells. SA-β-gal assays demonstrated that ectopic over-expression of miR-34a markedly increases IR-induced senescence, whereas knockdown of miR-34a by transfection with miR-34a inhibitors attenuates IR-induced senescence in H460 cells (Figures 3A & 3B). Similar results were observed in A549 cells (Figure 3C). These data suggest that miR-34a may play a critical role in modulating IR-induced premature senescence in human NSCLC cells.

**Figure 4:**
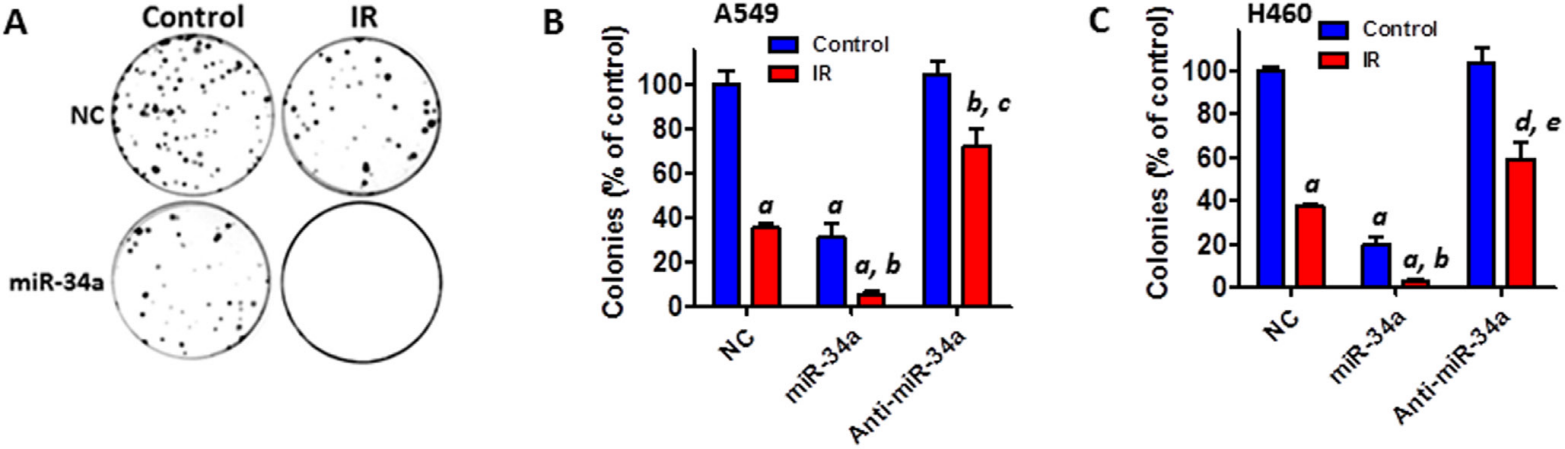
Transfection with miR-34a mimics augments IR-induced cell killing in NSCLC cells **(A)** Clonogenic assays were performed to determine the effect of miR-34a overexpression on IR-induced cell killing. Representative images of clonogenic assays of A549 cells are shown. **(B)** The number of colonies from irradiated (4 Gy) A549 cells was normalized to the percentage of colonies generated by control cells. **(C)** The number of colonies from irradiated (2 Gy) H460 cells was normalized to the percentage of colonies produced by control cells. All data are presented as mean ± SEM of three independent experiments. a, p < 0.001 vs. NC control; b, p < 0.01 vs. NC + IR; c, p < 0.05 vs. NC control; d, p < 0.01 vs. NC control; e, p < 0.05 vs. NC + IR.

Next, we determined the impact of miR-34a on IR-induced cell killing. Clonogenic survival assays revealed that transfection with miR-34a mimics significantly augments IR-induced cell death whereas inhibition of miR-34a by anti-miR-34a inhibitors diminishes IR-induced cell killing in both A549 and H460 cells (Figures 4A–4C). These results suggest that miR-34a mimics can be exploited as a new radiation sensitizer to enhance the efficacy of radiation therapy for human NSCLC.

### miR-34a modulates IR-induced senescence in NSCLC cells via targeting c-Myc

**Figure 5:**
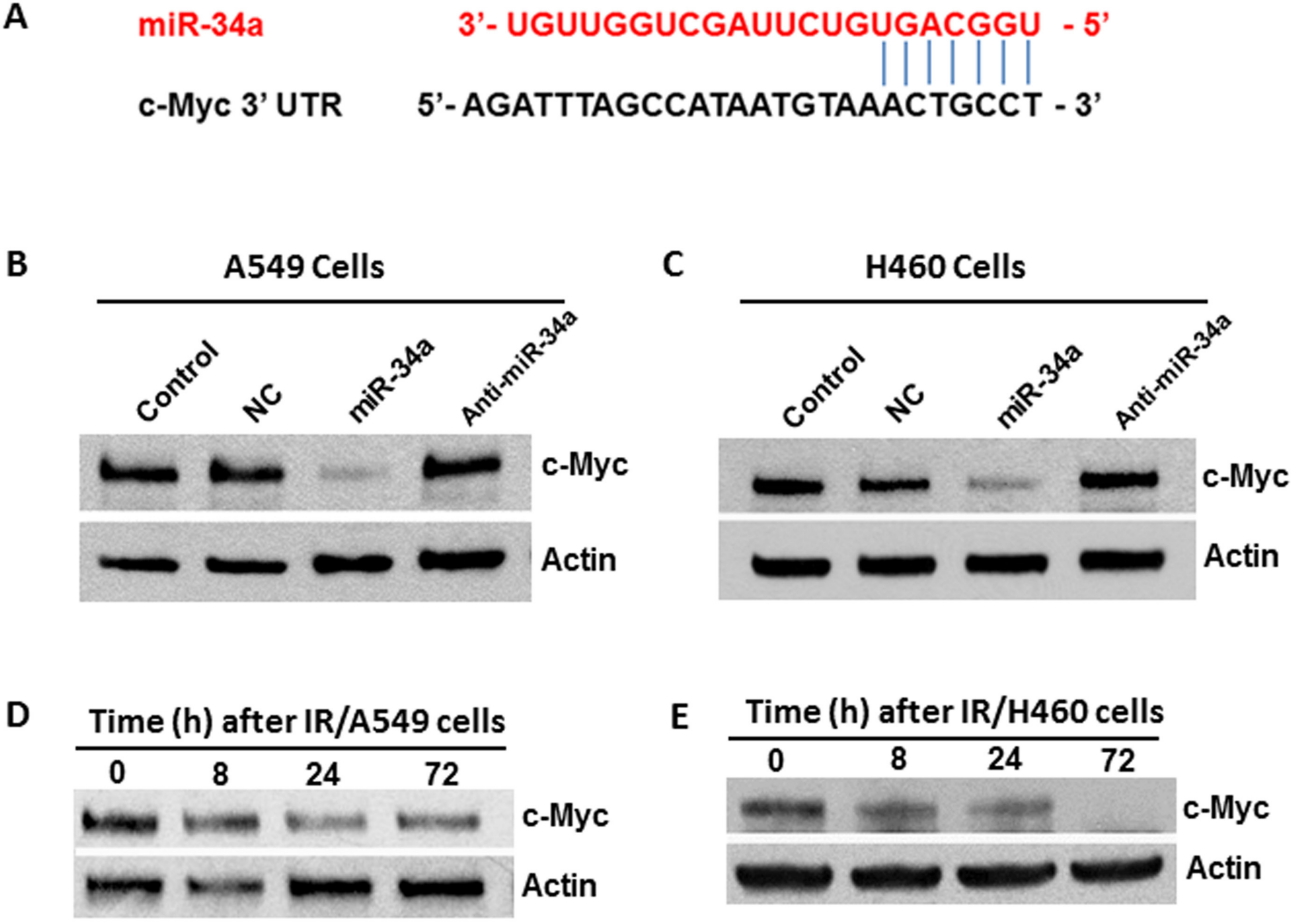
Myc is a target of miR-34a in human NSCLC cells **(A)** Schematic showing the predicted miR-34a binding site of c-Myc 3’-UTR. **(B)** A549 cells were transfected with miR-34a mimics (10 nM) or anti-miR-34a inhibitors (10 nM). An equal amount of NC miRNA was used as control. Myc expression was determined at 48 h after transfection using Western blot analysis. **(C)** H460 cells were transfected with miR-34a mimics or anti-miR-34a inhibitors using the same protocol as described in B. Myc expression was determined at 48 h after transfection using Western blot analysis. **(D-E)** Myc expression levels were determined in irradiated A549 and H460 cells at different time points after irradiation. β-actin was probed as a loading control.

To elucidate the mechanisms by which miR-34a modulates IR-induced senescence in NSCLC cells, we investigated potential target(s) of miR-34a that might be involved in regulating cellular senescence. Through bioinformatic analyses, we found that the seed sequence of miR-34a matches precisely with the 3’-untanslated region (3’-UTR) sequence of the *MYC* oncogene, suggesting that Myc is likely a target of miR-34a (Figure 5A). To experimentally validate this suggestion, we transfected A549 cells with synthesized miR-34a mimics and examined the impact of miR-34a overexpression on Myc expression. Western blot analyses revealed that transfection with miR-34a mimics resulted in a significant decrease in Myc expression, whereas no significant changes in Myc expression levels were observed in cells transfected either with NC miRNA or anti-miR-34a oligonucleotides (Figure 5B). Similar results were observed in H460 cells (Figure 5C). Taken together, these results demonstrate that Myc is a target of miR-34a in human NSCLC cells and suggest that miR-34a may modulate IR-induced senescence via targeting Myc. In agreement with this idea, our Western blot data showed that irradiation leads to a marked decrease of c-Myc expression in both A549 and H460 cells in a time-dependent manner (Figures 5D & 5E).

### Knockdown of Myc expression recapitulates the senescence-promoting effect of miR-34a

**Figure 6:**
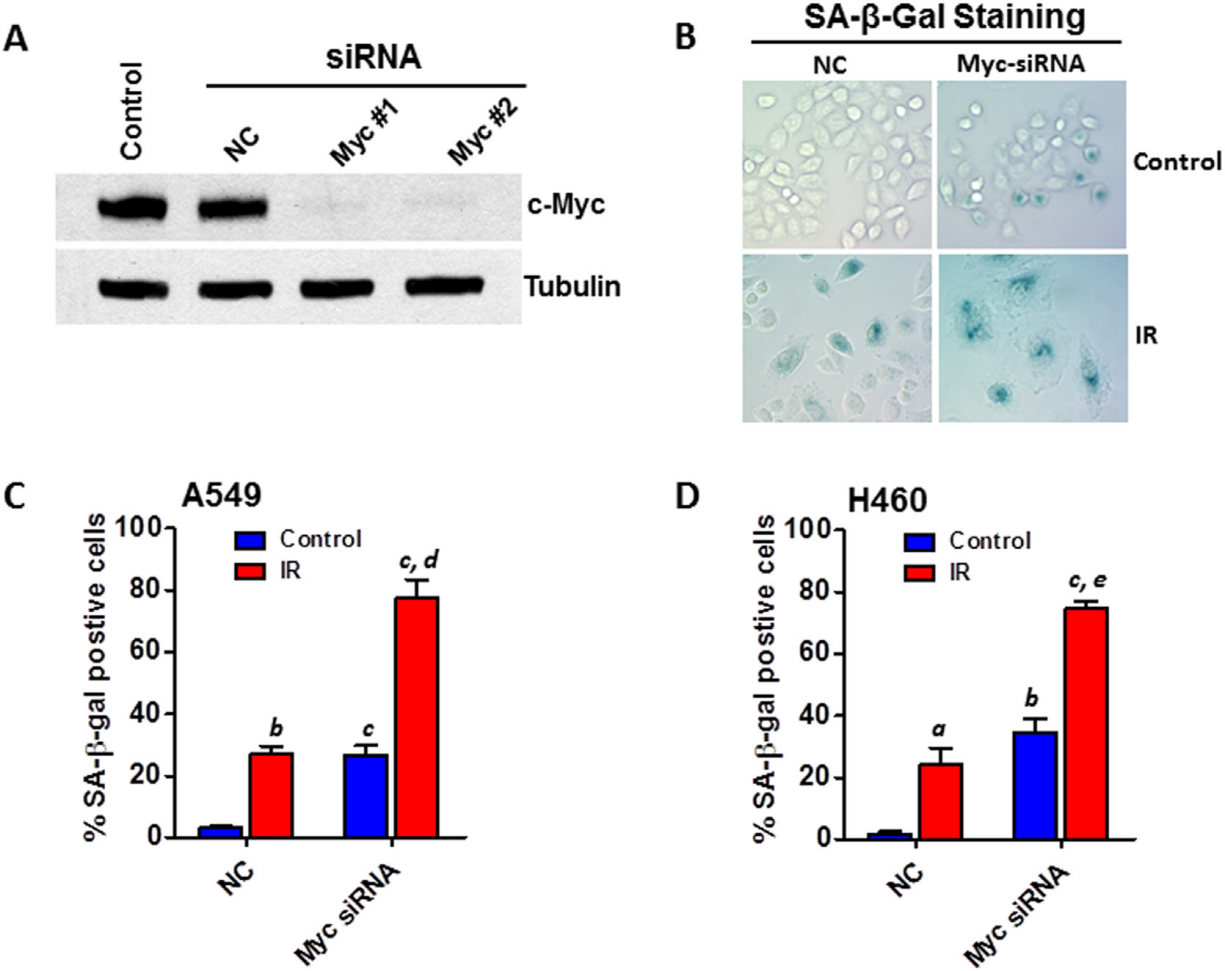
Knockdown of Myc expression recapitulates the senescence-promoting effect of miR-34a in NSCLC cells **(A)** A549 cells were transfected with Myc siRNA (20 nM) or an equal amount of non-targeting control siRNA (NC) as control using Lipofectamine RNAi MAX following the manufacturer’s protocol. The knockdown of Myc expression was confirmed 2 days after siRNA transfection by Western blot analysis. **(B)** SA-β-gal assays were performed to determine senescent cells 6 days after siRNA transfection with or without irradiation. Shown are representative images of SA-β-gal staining in H460 cells 6 days after irradiation (2 Gy). **(C)** The percentage of SA-β-gal positive senescent cells in irradiated (5 Gy) and non-irradiated (control) A549 cells is presented as mean ± SEM of three independent assays. **(D)** The percentage of SA-β-gal positive senescent cells in irradiated (2 Gy) and non-irradiated (control) H460 cells is presented as mean ± SEM of three independent assays. a, p < 0.05 vs. NC control; b, p < 0.01 vs. NC control; c, p < 0.001 vs. NC control; d, p < 0.01 vs. NC + IR; e, p < 0.001 vs. NC + IR.

To confirm the role of Myc in mediating miR-34a modulated IR-induced senescence, we investigated if knockdown (KD) of Myc expression can phenocopy the senescence-promoting effect of miR-34a. The KD of Myc expression by siRNA transfection was confirmed by Western blot analyses (Figure 6A). SA-β-gal staining analyses revealed that Myc KD significantly enhances IR-induced senescence in both A549 and H460 cells (Figures 6B–6D). These new data demonstrate for the first time that KD of Myc expression can recapitulate the senescence-promoting effect of miR-34a, which strongly support the hypothesis that miR-34a modulates IR-induced senescence in NSCLC cells via targeting Myc.

**Figure 7:**
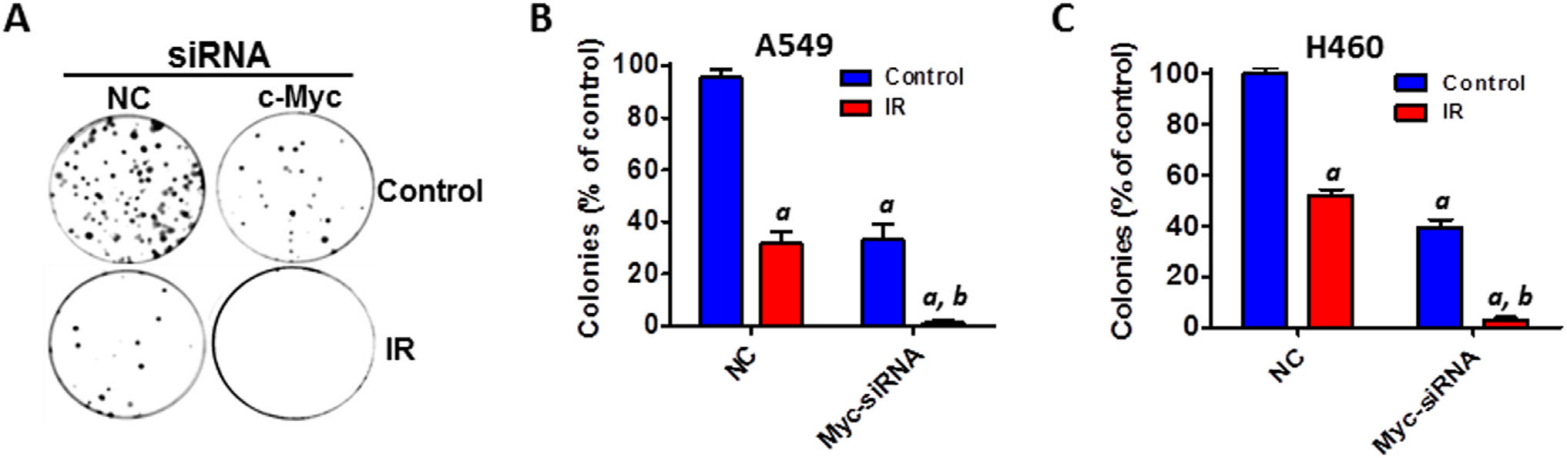
Silencing of Myc expression enhances IR-induced cell killing in NSCLC cells **(A)** A549 cells were transfected with Myc siRNA (20 nM) or an equal amount of non-targeting control siRNA (NC) as shown in Figure 6. The effect of Myc knockdown on IR-induced cell killing in NSCLC cells was determined by clonogenic assays at 48 h after siRNA transfection. **(B)** The number of colonies generated by irradiated A549 cells (4Gy) was normalized to the percentage of colonies produced by control cells. **(C)** The number of colonies generated by irradiated H460 cells (2Gy) was normalized to the percentage of colonies produced by control cells. Data are presented as mean ± SEM of three independent assays. a, p < 0.001 vs. NC control; b, p < 0.01 vs. NC + IR.

Furthermore, clonogenic assays were performed to determine the effect of Myc KD on IR-induced cell killing in NSCLC cells. The results showed that silencing of Myc by siRNA markedly enhances the cell killing effect of IR in both A549 and H460 cells (Figures 7A–7C). Collectively, these new findings have demonstrated for the first time that miR-34a enhances IR-induced cell killing and senescence induction in human NSCLC cells via targeting Myc.

## DISCUSSION

Cellular senescence is a state of permanent cell cycle arrest that can be triggered by continuous passage of cells in culture, oxidative stress, treatment with chemotherapeutic agents or radiation and activation of oncogenes such as Ras [5–11, 27–29]. Notably, it has been well-documented that the induction of senescence is an important mechanism whereby some chemotherapeutic agents as well as irradiation and cancer immunotherapies suppress tumor growth and arrest cancer progression [3, 5–12]. Our recent studies have demonstrated that radiation primarily induces premature senescence rather than apoptosis in human NSCLC cells [3]. Moreover, we have shown that pharmacological activation of p53 by Nutlin-3 enhances the tumor cell killing effect of radiation by promoting IR-induced premature senescence [3]. These observations suggest strongly that targeting of the senescence pathways may provide a novel and effective therapeutic strategy to improve the efficacy of lung cancer radiation therapy.

It is well known that many human tumors harbor various types of defects in apoptosis signaling pathways (e.g., loss of p53 and overexpression of BCL2) and as a results are resistant to apoptosis-based anticancer therapies [5, 30, 31]. In these cases, a senescence-targeted strategy is likely to be a more effective and practical therapy versus traditional apoptosis-inducing approaches [3, 5, 12, 30, 32]. In agreement with this idea, it has been shown that therapy-induced senescence can be achieved using much lower doses of chemotherapy than those required to induce apoptosis, suggesting that high doses of anticancer agent may lead to apoptosis whereas low level treatments primarily induce premature senescence in cancer cells [5, 6]. Compared to the traditional apoptosis-inducing strategies, this low dose approach can significantly reduce the side effects of anticancer therapy and thus improve the quality of life for cancer patients. Consistent with this concept, there is evidence indicating that cancer immunotherapies do not cause cytotoxic tumor cell death, but instead induce tumor cell senescence and thus arrest cancer progression [12, 33]. The major goals of this study were to characterize the role of miR-34a in modulating IR-induced premature senescence in human NSCLC cells and to determine if pharmacological up-regulation of miR-34a expression augments radiation-mediated tumor cell killing via promoting IR-induced senescence.

Recent studies from our and other labs have shown that miR-34a is involved in the regulation of cellular senescence [24, 34]. However, it was not known, until the present study, if miR-34a affects IR-induced premature senescence in human NSCLC cells. This study provides the first experimental evidence demonstrating that ectopic overexpression of miR-34a enhances the cell killing effects of irradiation by promoting IR-induced senescence in human NSCLC cells. These results suggest that treatment with synthesized miR-34a mimics may represent a new and effective therapeutic strategy to sensitize NSCLC to radiation therapy via promoting IR-induced premature senescence. Consistent with our findings, previous studies also showed that ectopic expression of miR-34a increases radiosensitivity of non-small cell lung cancer cells [35]. Notably, the present study is the first to demonstrate that IR induces a marked increase of miR-34a expression in human NSCLC cells in a dose- and time-dependent fashion. More importantly, we found that miR-34a modulates IR-induced senescence through targeting the c-Myc oncoprotein and that knockdown of c-Myc expression copies the phenotype of miR-34a mimic transfection. These new findings provide a novel insight into the mechanisms by which miR-34a enhances IR-induced senescence in human NSCLC cells.

The *MYC* oncogene encodes for a transcription factor that is overexpressed in multiple human cancer types, including NSCLC [36–40]. It has been well-documented that various tumors are dependent on Myc and that inactivation of Myc leads to tumor regression in multiple preclinical tumor models [32, 41, 42]. Induction of senescence is an important mechanism of tumor prevention in response to oncogene activation [43, 44]. Previous studies have suggested that Myc may promote tumorigenesis through antagonizing oncogene activation-induced senescence [27, 28, 32, 45], indicating a critical role for Myc in modulating cellular senescence. However, the role of Myc in IR-induced tumor cell senescence is poorly understood. In this study, we have demonstrated for the first time that silencing of Myc expression markedly enhances IR-induced senescence in NSCLC cells. This new observation suggests that targeted inhibition of Myc can be exploited as a new approach to improve the efficacy of NSCLC radiotherapy via enhancing IR-induced senescence. Consistent with this suggestion, it has been shown that inactivation of MYC led to tumor regression *in vivo* through the induction of senescence in hepatocellular carcinoma and lymphoma cells [32]. In addition, we found that knockdown of Myc exhibits a similar degree of senescence-promoting effect compared with miR-34a mimic transfection, suggesting that Myc is likely the key target of miR-34a involved in mediating the senescence-enhancing activity of miR-34a, particularly in human NSCLC cells.

Although we and others have shown that miR-34a is an important player in regulating cellular senescence [24, 34], the mechanisms whereby miR-34a enhances IR-induced senescence in NSCLC cells have not been well understood. Here we show that Myc is a putative target of miR-34a in human NSCLC cells. More importantly, our studies have revealed that knockdown of Myc expression recapitulates the senescence-promoting effects of miR-34a and augments IR-induced cell killing in NSCLC cells. These new findings demonstrate for the first time that pharmacological treatment with synthetic miR-34a mimics augments the cell-killing effect of irradiation through promoting senescence induction in human NSCLC cells via targeting Myc. Collectively, the results of this study provide a strong rational for further preclinical studies and potential clinical trials for utilizing synthetic miR-34a mimics as a novel radiation sensitizer to improve the efficacy of lung cancer radiotherapy.

## MATERIALS AND METHODS

### Cell lines and culture

Human non-small cell lung cancer (NSCLC) cell lines A549 and H460 were purchased from American Type Culture Collection. A549 cells were cultured in DMEM medium containing 10% FBS, 2 mM L-glutamine and 100 microgram/ml of penicillin-streptomycin (Invitrogen). H460 cells were grown in RPMI-1640 medium containing 10% FBS, 2 mM L-glutamine and 100 microgram/ml of penicillin-streptomycin.

### TaqMan miRNA assays

Expression levels of miRNAs were determined using TaqMan miRNA assays as previously described [24]. Briefly, RNAs were extracted from human NSCLC cells using a mirVana™ miRNA Isolation Kit (Ambion, Austin, TX) following the manufacturer’s protocol. Quality and quantity of RNA samples were assessed using a Nanodrop spectrometer. RNA samples were reverse transcribed using a TaqMan MicroRNA Reverse Transcription Kit (Applied Biosystems) and a set of stem loop miRNA-specific primers (Applied Biosystems). Real-time PCR assays were performed using a LightCycler^®^ 480 System (Roche). The expression levels of miR-34a were calculated and normalized to those of endogenous U6 miRNA using the C_T_ method as we have reported previously [24].

### miRNA mimic transfection

The synthesized miRNA mimics and inhibitors were purchased from Qiagen (Valencia, CA). Ectopic overexpression of miR-34a was achieved by transfection of human NSCLC cells with miR-34a mimics. Knockdown of miR-34a was performed using synthetic miR-34a inhibitors (miR-34a antisense oligonucleotides) employing Lipofectamine RNAi MAX (Invitrogen) according to the manufacturer’s protocol. An aliquot of the cells was simultaneously transfected with negative control (NC) miRNA mimics as control.

### Radiation and clonogenic survival assay

A549 and H460 cells were transfected with miR-34a mimics or c-Myc-specific siRNA using Lipofectamine RNAi MAX (Invitrogen) according to the manufacturer’s protocol. At 48 h after transfection, cells were irradiated with a ^137^Cs irradiator. Irradiated and non-irradiated control cells were cultured in 60 mm dishes for 10 – 12 days to allow formation of cell colonies. The colonies were fixed and stained with 0.5% crystal violet (Sigma) in methanol for 30 min. The number of colonies (≥ 50 cells) was scored and photographed as described previously [3, 10].

### BrdU incorporation and confocal microscopy

A549 and H460 human NSCLC cells were cultured in slide chambers and labeled with BrdU using a BrdU labeling kit (BD Biosciences) according to the manufacturer’s instructions. Slides were blocked with 5% normal goat serum for 30 min prior to incubation with a mouse anti-BrdU monoclonal antibody (1:200, Sigma) overnight at 4°C. After intensive washes, cells were incubated with Alexa Fluor-594 conjugated goat anti-mouse IgG (1:200, Invitrogen) for 1h at room temperature and nuclei were counterstained with DAPI. Finally, slides were imaged and analyzed using a Zeiss LSM 880 laser scanning confocal microscope (Carl Zeiss, Oberkochen Germany).

### siRNA transfection

To knockdown Myc expression, A549 or H460 cells were transfected with Myc-specific siRNAs (Qiagen, Valencia, CA) using Lipofectamine RNAi MAX (Invitrogen) according to the manufacturer’s protocol. AllStars negative control (NC) siRNAs (Qiagen, Valencia, CA) were used as control. At 48 h after transfection, levels of Myc expression in siRNA transfected NSCLC cells were assessed by Western blot analyses.

### Senescence-associated β-galactosidase (SA-β-gal) staining

*In situ* staining of SA-β-gal was performed using a senescence β-galactosidase staining kit (Cell Signaling) to determine senescent cells as we have reported previously [3, 6, 46].

### Western blot analysis

Western blot analyses were performed as previously described [47]. Briefly, protein samples were extracted using cell lysis buffer (Cell Signaling) supplemented with a cocktail of proteinase inhibitors (Sigma). The protein concentrations were quantified using the Bio-Rad Dc protein assay kit (Bio-Rad Laboratories, Hercules, CA). Fifty microgram protein samples were resolved on 4 - 20% Mini-Protean TGX gels (Bio-Rad) and transferred onto 0.2 μM PVDF membrane (Millipore). Blots were blocked with 5% non-fat milk for 1-2 hours at room temperature and then probed with primary antibodies and incubated at 4°C overnight. After extensive washing with TBS-T, blots were incubated with appropriate HRP-conjugated secondary antibody for 1.5 h at room temperature. Protein bands were detected using an ECL Plus Western Blotting Detection System (GE Healthcare Life Science).

### Statistical analysis

Comparisons between two groups were carried out using Student’s *t*-test. Differences were considered statistically significant at *p* < 0.05. All analyses were carried out with the GraphPad Prism program (GraphPad Software, Inc. San Diego, CA).

## SUPPLEMENTARY MATERIALS FIGURE


