# Supplementary material for: MiR-34a modulates ionizing radiation-induced senescence in lung cancer cells
Source: Oncotarget. 2017 Jul 15;8(41):69797–807. doi: 10.18632/oncotarget.19267 (PMC5642517; doi:10.18632/oncotarget.19267)
Supplement: Supplementary file 1 [file oncotarget-08-69797-s001.pdf]

# MiR-34a modulates ionizing radiation-induced senescence in lung cancer cells

## SUPPLEMENTARY MATERIALS

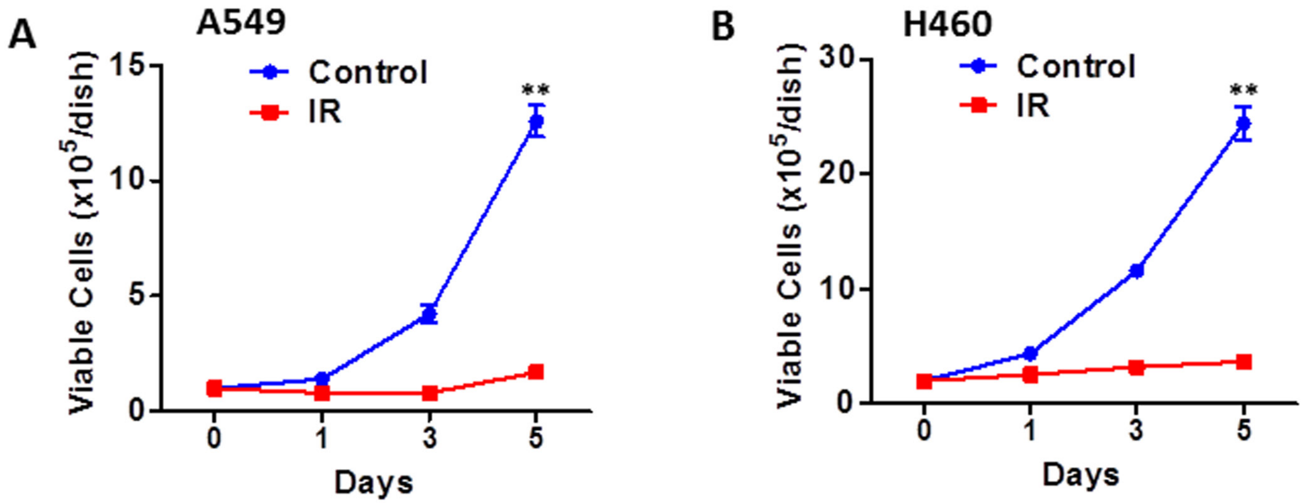


**Supplementary Figure 1: Irradiation arrests the growth of human NSCLC cells.** A549 cells **(A)** were exposed 10 Gy irradiation while H460 cells **(B)** were treated with 5 Gy IR. Changes in cell number for each group were monitored and plotted versus days of culture. The results show that irradiated A549 and H460 cells lose their ability to proliferate while the control cells grow exponentially. ** $p < 0.01$ compared with control cells.
